# Observational study of effects of pharyngeal stimulation by carbonated solution on repetitive voluntary swallowing in humans

**DOI:** 10.1097/MD.0000000000034889

**Published:** 2023-08-25

**Authors:** Mika Tsuchiya, Yumiko Kubo, Naomi Maruyama, Chie Omori, Hideyuki Fukami

**Affiliations:** a Department of Oral Health Sciences, Faculty of Nursing and Health Care, Baika Women’s University, Ibaraki, Osaka, Japan.

**Keywords:** carbonation, pharyngeal region, sensory input, swallowing

## Abstract

In this study, we conducted observational study to examine the effects of pharyngeal stimulation by a bolus of carbonated solution on repetitive voluntary swallowing in humans.

Twelve healthy participants had a fine silicone tube inserted into their pharyngeal region, through which various solutions were slowly infused (0.2 mL/minute) to stimulate the pharyngeal mucosa without activating mechanoreceptors. The solutions included 0.3M NaCl (NaCl), carbonated 0.3M NaCl (NaCl + CA), 0.3M NaCl with acetic acid, distilled water, and carbonated distilled water. We used NaCl to inhibit water-sensitive neurons in the pharyngeal mucosa and enable the evaluation of the effects of carbonic acid stimulation on swallowing. Participants were instructed to repeat swallows as rapidly as possible during the infusion, and the swallowing interval (SI) was measured via submental surface electromyographic activity.

SI was significantly shorter during the infusion of NaCl + CA, distilled water, and carbonated distilled water than during the infusion of NaCl. There was a significant positive correlation between SI with NaCl stimulation and the facilitative effects of the other solutions. Longer SIs with NaCl stimulation indicated potent facilitative effects. Thus, stimulation with NaCl + CA facilitated swallowing by reducing SI. Furthermore, the facilitative effects of SI were more pronounced in participants who had difficulty with repetitive voluntary swallowing.

The sensation induced by carbonated solution may enhance the ability for repetitive voluntary swallowing, making it a potentially useful approach for rehabilitating patients with dysphagia.

## 1. Introduction

Swallowing depends on activation of central and peripheral nervous control systems by different feeding and swallowing phases.^[1]^ The peripheral nervous system includes sensory information from the oropharyngeal region. Sensory information is essential for neurogenic regulation of swallowing. Excitation of sensory receptors in the oropharyngeal mucosa activates the trigeminal, glossopharyngeal, and vagus nerves. These nerves converge at the swallowing central pattern generator in the medulla oblongata.^[2]^ This sensory input influences swallowing, modulates motor output, and activates ascending pathways.^[3,4]^ Sensory input from the oropharyngeal region is critical in facilitating swallowing, and many human and animal experiments have demonstrated that sensory information from the oropharyngeal region triggers swallowing and modulates swallowing-related motor activity.^[3]^ Especially in humans, the effect of sensory stimulation on swallowing has been studied from the perspective of rehabilitation of dysphagia. Therefore, it has been confirmed that sensory effects of cold mechanical stimulation,^[5,6]^ bolus volume or viscosity modification,^[7,8]^ and chemical stimulation^[9–12]^ on swallowing.

Carbonated water applied to the mouth produces various sensory qualities. For example, oral stimulation by carbonated water causes pungent and tactile perception by activating thermal, chemogenic, and mechanical receptors.^[13,14]^ Therefore, the effects of carbonation on swallowing have also been investigated in healthy and dysphagic people.^[15–21]^ However, there is disagreement among studies regarding the effects of sensory information induced by bolus carbonation. Min, et al^[17]^ showed that carbonated water significantly decreased the swallowing onset time and increased the swallowing-related electromyography (EMG) activity amplitude in a carbonation concentration-dependent manner in healthy subjects. Conversely, 5 mL carbonated boluses had no significant effect on the amplitude or duration of swallowing-related submental EMG activity.^[15]^ This conflicting effect of bolus carbonation on swallowing may be due to the nature of the bolus itself.

Removal of other sensory influences is necessary to examine the stimulating effects of a carbonated bolus. Two sensory influences must be considered when investigating the sensory effect of carbonation on swallowing: the volume of the bolus and the stimulating effect of water. Previous reports have indicated that an increase in bolus volume elicits an increase in tongue propulsive forces and shortens the latencies to evoke swallowing.^[7,22]^ An increased bolus volume may affect swallowing activity by stimulating mechanoreceptors in the pharyngeal region. CO_2_ gas is commonly dissolved into water to investigate the effects of carbonation on swallowing. The effectiveness of water application to the pharyngolaryngeal region on swallowing has been demonstrated in both animal and human studies. Water application to the larynx initiates the swallowing reflex by excitation of water-sensitive fibers in the superior laryngeal nerve, which is a branch of the vagus nerve, and excitation of water-sensitive fibers within the superior laryngeal nerve has been shown to be inhibited by application of hypertonic 0.3M NaCl solution (NaCl) solution in the rabbit.^[23]^ Yahagi, et al^[12]^ investigated the effect of applying water and NaCl solution to the pharyngeal region on voluntary swallowing in humans. To minimize excitation of mechanoreceptors, they applied water and NaCl solution to the pharyngeal region at a very slow infusion speed (0.2 mL/minute) by orally inserting a fine tube. Their results indicated that water application shortened the swallowing interval (SI) during repetitive voluntary swallowing compared with NaCl solution application. These findings imply that the human pharynx contains nerve fibers that are sensitive to water, and that the infusion of NaCl results in the inhibition of excitation of these fibers, leading to the prolongation of swallowing intervals. Yahagi et al^[12]^ suggested that the SIs in NaCl administration indicates the central swallowing capacity of the subject as there is no sensory facilitation from peripheral, and that sensory information from water-sensitive nerve fibers excited by water compensates for this central swallowing capacity.

Given the presence of mechanoreceptors and water-sensitive fibers in the oral and pharyngeal mucosa of human beings, the ingestion of a substantial volume of carbonated water may potentially stimulate mechanoreceptors and water-sensitive fibers. However, previous studies have found it challenging to isolate the effect of carbonic acid stimulation on swallowing due to the presence of other sensations. By inhibiting the excitation of these pharyngeal water-sensitive nerve fibers and mechanoreceptors, it would be possible to assess the effect of carbonic acid alone on swallowing, and to resolve the inconsistencies in the previously reported effects of carbonic acid on swallowing. One-way to achieve this is by infusing a NaCl solution at a very slow rate directly into the pharyngeal region to minimize the excitation of these sensory receptors in the pharyngeal region. By injecting carbonated NaCl solution into the pharynx at a slow infusion rate, it is possible to study the effects of carbonic acid stimulation on swallowing without exciting the water-sensitive neurons and mechanoreceptors present in the pharynx. As pharyngeal hypesthesia plays a crucial role in the development of dysphagia,^[24]^ investigating the physiological mechanisms underlying the facilitation of swallowing via pharyngeal sensory stimulation by carbonation may lead to the development of novel approaches to swallowing rehabilitation. Thus, we hypothesize that carbon dioxide-sensitive neurons exist in the pharyngeal region, and only the effect of bolus carbonation in facilitating swallowing can be investigated using carbonated NaCl solutions. Moreover, if carbonic acid stimulation of the pharyngeal region is effective for voluntary repetitive swallowing, the SIs should be shorter than that of NaCl stimulation. Therefore, we conducted observational study to investigate the effect of carbonation on the modulation of repetitive voluntary swallowing by measuring SIs during the administration of carbonated NaCl solution to the pharyngeal region.

## 2. Materials and Methods

Twelve healthy volunteers (5 men and 7 women; mean age, 37 years; range, 21–59 years) who had no oropharyngeal disorders, were taking no medication, and had no impairment of taste or olfaction were enrolled in this observational study. All subjects were recruited from university staff and students and enrolled by researchers. Subjects were instructed to fast and drink no more than 1 hour before the experiment. The experiment was conducted in the Department of Oral Health Sciences laboratory. Subjects were assigned to 1 group by researchers, and all received the same experimental treatment. Although the subjects were not blinded in this experimental system, they were not told which liquids they were swallowing during the experiment. The minimum required sample size has been calculated using the G*Power software version 3.1.9.7 (Heinrich Heine University, Dusseldorf, Germany). Effect size = 0.4, α error probability = 0.05, and power of 90% were used. Consequently, the calculation gave a required number of 11 in total. The study protocol was approved by the Ethics Committee of Baika Women’s University.

Stimulating solution was applied to the pharyngeal region through a fine silicone tube of 1-mm outer diameter (94-0451-4; Sansyo, Tokyo, Japan) that was orally inserted into the throat. The stimulating solutions used in this study were distilled water (DW), carbonated distilled water (DW + CA), NaCl, carbonated 0.3M NaCl solution (NaCl + CA), and pH-adjusted 0.3M NaCl solution (NaCl + AA). We chose 0.3M NaCl solution because of its inhibitory effects on the excitation of water-sensitive fibers. Because carbonation changes the solution pH, we adjusted the pH of the 0.3M NaCl solution by adding acetic acid to achieve a pH of 3.2. The investigators prepared the carbonated solution prior to each infusion by adding CO_2_ from a canister to 100 mL of DW or NaCl solution in a commercially available soda maker (DRM 1005; Drinkmate, Ann Arbor, MI). All solutions were used at room temperature (20ºC). The tip of the tube was positioned at a distance of 12 cm from the mandibular incisors; this distance corresponds to the position of the pharyngeal region.^[10,12]^ Infusion of a small amount of solution into the tube at this tip position did not give rise to any taste, burning, stinging, tingling, numbness, or tactile sensation. To minimize the mechanical effect of infusion, solutions were delivered through the tube using an infusion pump (SP100i; World Precision Instruments, Sarasota, FL) at a very slow infusion rate (0.2 mL/minute). The order of the solutions was randomized by using dice and random numbers. The participants were not informed of the solution being administered. During the experiment, each participant sat upright on a chair. They were instructed to perform repetitive swallowing as rapidly as possible after the onset of infusion. Before the experimental sessions, a 2-minute training session was performed. The participants swallowed infused DW during training session. During each infusion, repetitive swallowing was performed for about 3 minutes. Between trials, the participants were told to drink water as they wished, and the next stimulation was performed after an interval of 7 minutes. Surface EMG was recorded from the left suprahyoid muscles to examine the muscle activity associated with swallowing. Two silver/silver chloride electrodes were taped under the chin, and surface EMG was recorded with a biological signal acquisition system (PowerLab 26T; ADInstruments Japan Inc., Nagoya, Japan). The participants pressed a button connected to the PowerLab system immediately after swallowing to record the actual swallowing time point. The SI was measured between 2 consecutive marks indicated by pressing the button. EMG burst activity confirmed that the participant was actually swallowing when the button was pressed. The first five SIs were discarded because of the potential influence of residual saliva on the SI. The average of the next five consecutive SIs was taken as the participant’s SI.

Yahagi, et al^[12]^ reported that applying NaCl solution to the pharynx at a very slow infusion rate suppresses the excitation of water-sensitive nerve fibers and mechanoreceptors in the human pharynx. Therefore, they subtracted the SI with water from the SI with NaCl solution to evaluate the facilitative effects of water on swallowing. In the present study, we subtracted the SI with each solution from the SI with NaCl solution to evaluate the facilitative effects of water, carbonation, and acid sensation on swallowing.

Statistical analysis was conducted using SPSS version 26 (IBM Corp., Armonk, NY). Repeated-measures analysis of variance was performed, and the level of significance was set at *P* < .05. Values are presented as mean ± standard error of the mean.

## 3. Results

**Figure 1.**
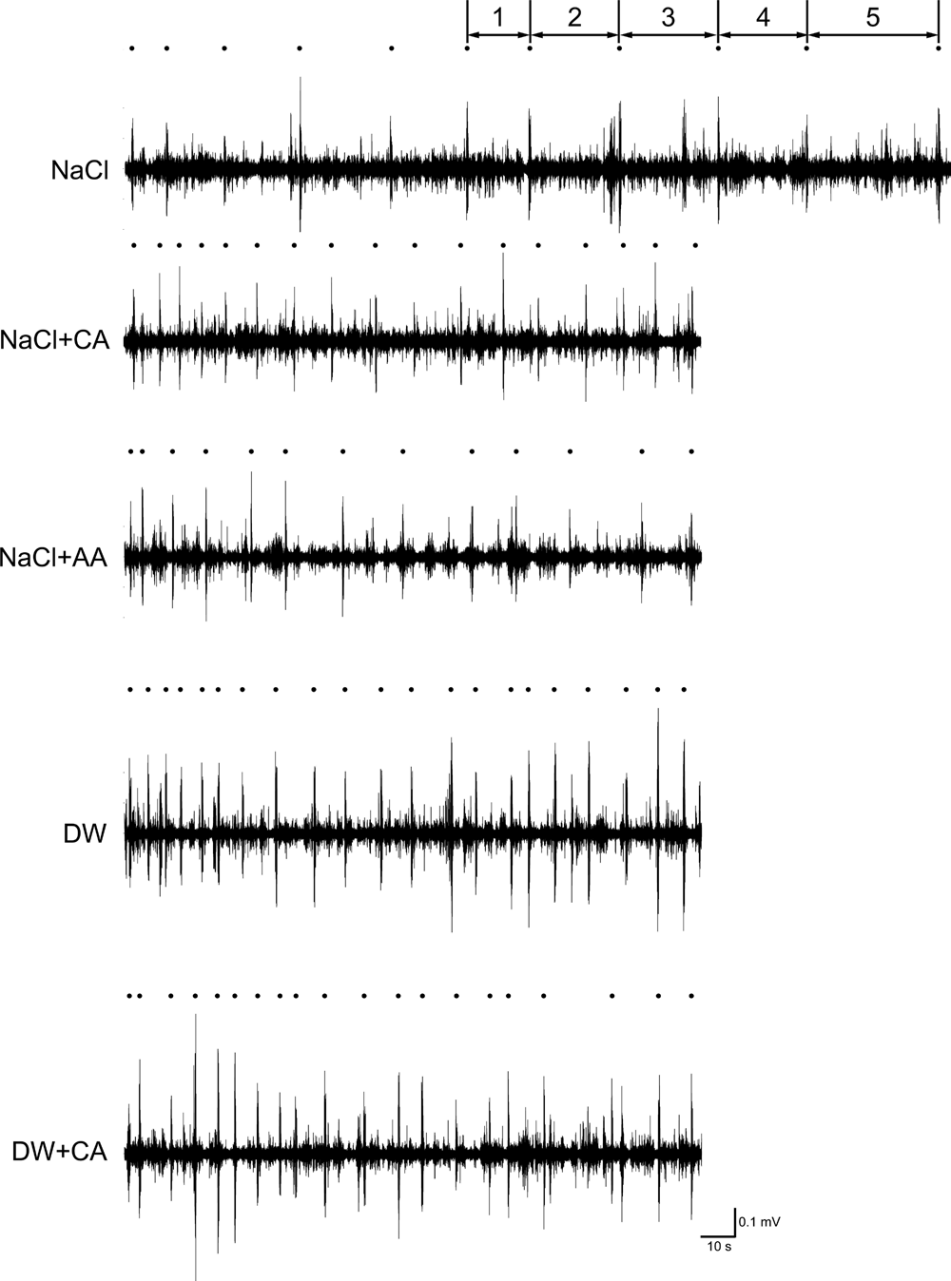
Example of submental surface EMG recordings during repetitive voluntary swallowing with infusion of stimulating solutions. The dots above each EMG recording indicate the actual swallowing time points. To reduce the influence of residual saliva, the SI was obtained by an average of five continuous SIs (1–5). The first five SIs after the beginning of swallowing were discarded to reduce the influence of residual saliva on the SI. EMG = electromyography, SI = swallowing interval.

**Figure 2.**
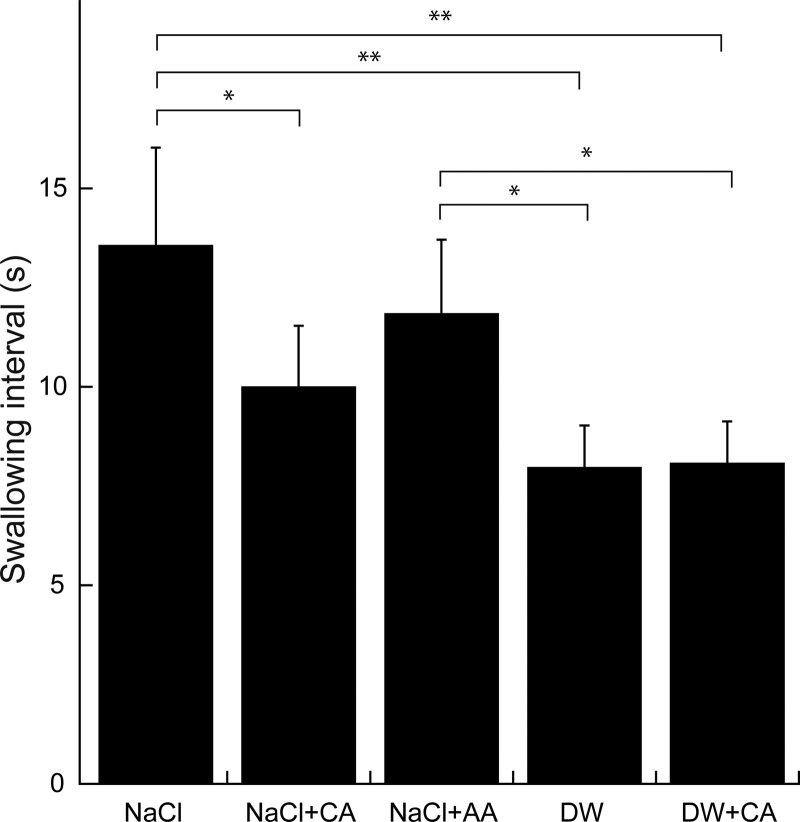
Effects of infusion of solutions on the SI. *P < .05, **P < .001. SI = swallowing interval.

Data obtained from all subjects were analyzed. Figure 1 shows typical EMG recordings of the suprahyoid muscles and actual swallowing points during repeated voluntary swallowing at the maximum frequency with infusion of the solution into the pharyngeal region at a low infusion rate. Infusion of NaCl + CA, DW, and DW + CA resulted in a relatively shorter SI than did infusion of NaCl and NaCl + AA. Figure 2 shows the effects of each stimulating solution on the SI. NaCl + CA, DW, and DW + CA resulted in a short SI, whereas 0.3M NaCl and NaCl + AA resulted in a prolonged SI. NaCl + CA produced a significantly shorter SI than did NaCl. Although NaCl + CA produced a shorter SI than did NaCl + AA (11.17 vs 12.96 seconds, respectively), the difference was not statistically significant.

**Figure 3.**
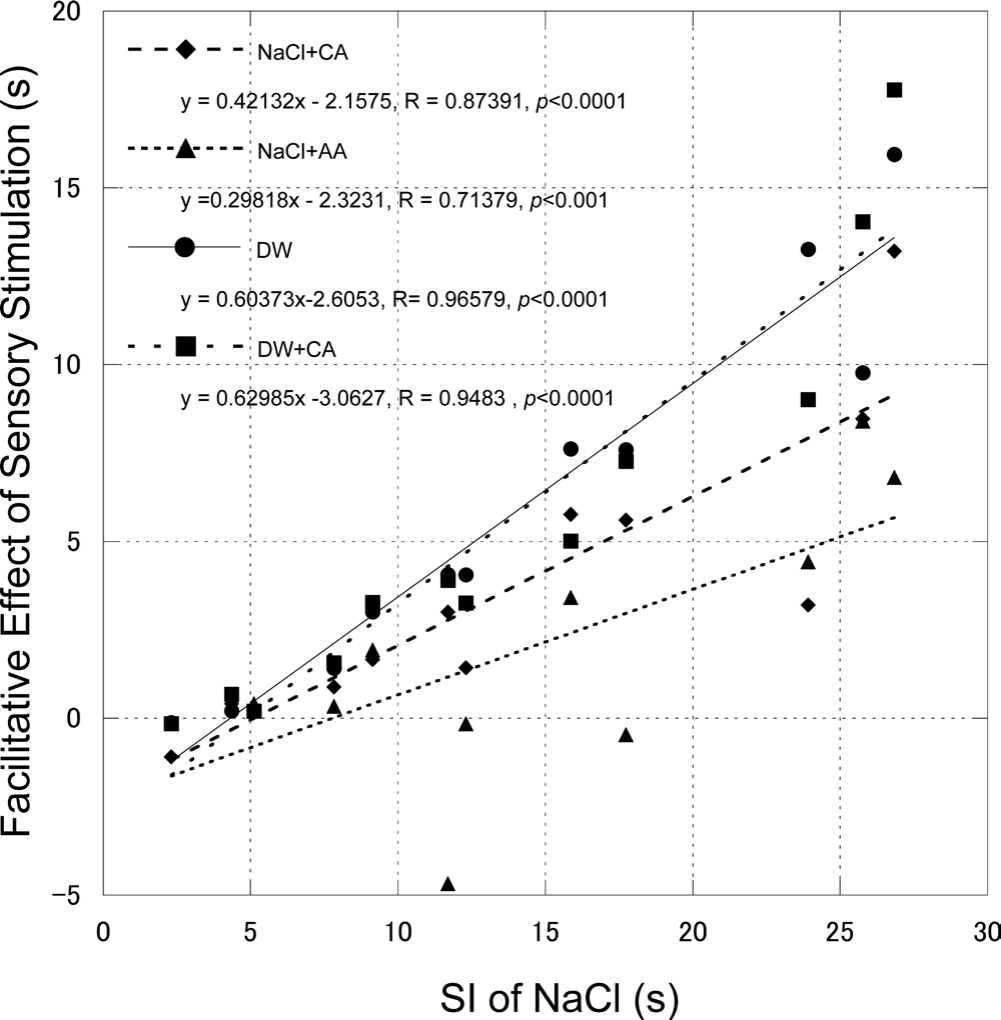
Correlation between the SI with 0.3M NaCl and the facilitating effect of sensory stimulations. The facilitative effects were defined by subtracting each participant’s SIs with NaCl + CA, NaCl + AA, DW, and DW + CA from the SIs with NaCl. There were significant positive correlations between the SI of NaCl and each facilitative effect. DW = distilled water, DW + CA = carbonated distilled water, NaCl = 0.3M NaCl solution, NaCl + AA = pH-adjusted 0.3M NaCl solution, NaCl + CA = carbonated 0.3M NaCl solution, SI = swallowing interval.

For all stimulant solutions, the SI showed great interindividual variation (NaCl: 4.99–25.77 seconds, NaCl + CA: 3.97–19.79 seconds, NaCl + AA: 4.41–19.06 seconds, DW: 4.32–17.47 seconds, and DW + CA: 3.7–16.15 seconds). Similarly, the facilitative effects of water, carbonic acid, and acid showed wide variation among the participants. However, significant positive correlations were observed between the SI with NaCl and the facilitative effects of the other solutions (Fig. 3). It was found that participants with longer SI when applying NaCl solution demonstrated potent facilitative effects.

## 4. Discussion

This study demonstrated the facilitative effect of initiation of repetitive voluntary swallowing by bolus carbonation. Compared with the application of NaCl, the application of NaCl + CA indicated a significantly shorter SI. However, a significant difference in the SI was not observed between DW and DW + CA. Pharyngeal hypesthesia constitutes a crucial factor in the development of dysphagia.^[24]^ Injecting NaCl slowly into the pharyngeal region can be considered as an approach to simulate the reduced sensation in the pharynx. The very slow infusion of NaCl + CA solution minimized mechanical and water sensory information from the pharyngeal mucosa, allowing for the appearance of the sensory effect of carbonation on swallowing. And, it is possible to evaluate the usefulness of pharyngeal carbonic acid stimulation as a novel rehabilitation modality for patients with dysphagia.

This paper presents the initial evidence indicating that carbonic acid stimulation confined to the pharyngeal region facilitates voluntary repetitive swallowing. The mechanism of how carbonic acid stimulation in the pharyngeal region is perceived and facilitates swallowing remains unclear. Whether the sensation elicited by carbonated water is of a mechanical or chemogenic origin has been debated. There is some evidence that the sensation evoked by carbonated water is chemogenic. Applying carbonated water to the tongue activates lingual nociceptors by converting CO_2_ to carbonic acid in a reaction catalyzed by carbonic anhydrase. An animal study showed that sour receptor-expressing taste cells on the tongue act as cellular sensors for carbonation.^[25]^ The study showed that PKD2L1-expressing sour-sensing cells mediate the taste response to carbonation and that taste responses to carbonation require carbonic anhydrase 4. In addition to the sour receptor PKD2L1, many other receptors are known to act as acid receptors. TRPV1 and acid-sensing ion channels, 2 types of acid sensors, are reportedly present in the human oropharynx.^[26,27]^ This suggests that the sensation in the oropharyngeal region induced by carbonation occurs by stimulation of acid sensors. In the present study, however, the matched-acidity solution (NaCl + AA) showed no significant facilitative effects on shortening the SI in comparison to NaCl. Wise, et al^[28]^ reported the influence of bubbles on the perception of carbonation bite. Innocuous tactile sensation induced by bubbles can enhance chemogenic sensation. Therefore, the shortening effects of the SI by carbonated NaCl observed in the present study might be explained by mechanical stimulation by bubbles. However, because there was no significant shortening effect on the SI between carbonated NaCl and NaCl + AA, the present results do not rule out the possibility of an acid effect on SI shortening. Another plausible explanation is the direct sensing of CO_2_. Earlier studies have indicated that the activation of temperature-sensitive transient receptor potential channels can augment the swallowing reflex.^[29]^ Applying TRPA1channel agonists, such as piperine,^[30]^ cinnamaldehyde and citral^[31]^ has been shown to improve the swallowing response in dysphagia patients. It has also been reported that CO_2_ activates TRPA1.^[32]^ TRPA1 is distributed in the laryngopharyngeal region, and pharyngeal TRPA1s act as chemosensors that trigger the swallowing reflex in rats.^[33]^ Stimulation of CO_2_ induced from carbonic acid may activate pharyngeal TRPA1s and facilitate repetitive swallowing by triggering the swallowing reflex.

Several recent studies have shown the effects of carbonation on swallowing in healthy subjects.^[17,21,34]^ These studies revealed that carbonation increased swallowing-related muscle activities (orbicularis oris, masseter, suprahyoid, and infrahyoid) and decreased the swallowing onset time, swallow velocity, and volume per swallow. However, the present study did not find a significant effect of DW + CA on the shortening of the SI compared to DW. Several factors may be associated with this discrepancy. One potential factor is the presence of methodological differences between the studies. In previous studies, the carbonated solution was applied to the mouth, and the subjects swallowed the solution,^[17,21,34]^ which included both the oral and pharyngeal phases of swallowing. In contrast, we applied the carbonated solution directly to the pharynx, including only the pharyngeal phase in our experimental condition. Min, et al^[17]^ found that carbonation affects the orbicularis oris and masseter muscle activities more strongly than the submental and infrahyoid muscle activities and concluded that the effect of carbonation on EMG amplitude was greater in the oral phase than in the pharyngeal phase. Therefore, the relatively weak effects of carbonation on swallowing-related behavior in the pharyngeal phase in the present study may explain the lack of a significant facilitative effect on the SI between DW and DW + CA. Another potential factor is the presence of interindividual differences in swallowing ability. As shown in Figure 3, a longer SI during application of NaCl solution indicated potent facilitatory effects by sensory stimulation. Previous studies have shown that sensory information from the pharyngeal region compensates for the difficulty in performing repetitive voluntary swallowing by modulating the central neural systems of swallowing.^[10,12]^ The facilitative effects of pharyngeal water application on swallowing can be strong. Therefore, in participants who had a relatively short SI with NaCl, the stimulating effect of carbonated water may have been masked by sufficient compensation from water stimulation. Participants with longer SIs when applying NaCl (>25 seconds) tended to show more potent facilitatory effects for DW + CA than for DW (Fig. 3).

The CO_2_ concentration in water depends on temperature. Compared with the samples at 3°C, the CO_2_ loss was 9% greater in the 16°C samples and 21% greater in the 22°C samples.^[14]^ Cold stimulation of the oropharyngeal region is well known to affect swallowing-related activities.^[16,18,35]^ Therefore, to reduce the effect of cold temperature on swallowing, stimulus solutions were set at 20°C in this study. Yau and McDaniel^[14]^ reported that the perception of carbonation intensity was higher at a lower temperature than at a higher temperature. Green^[13]^ indicated that carbonated water induced burning, stinging, tingling, numbness, and tactile sensations. The author reported that burning and stinging sensations were increased at lower temperatures (4°C) than at higher temperatures (24°C), but temperature did not affect tingling, numbness, or tactile sensations. Takeuchi, et al^[21]^ investigated the effect of temperature (5°C, 15°C, and 20°C) and carbonation of water on swallowing behavior (volume of solution swallowed, ease of holding the solution in the mouth, and ease of swallowing). They showed that carbonated water significantly affected swallowing behavior; however, the temperature had no significant interaction. Therefore, we assume that the 20°C carbonated solutions used in the present experiment provided sufficient carbonated stimulation of the pharynx and shortened the SI. Although pharyngeal stimulation with a carbonated solution at 20°C produces insufficient sensory information to recognize sensory quality, it may provide sensory information to shorten the SI.

Several limitations of this study must be considered. First, our study exclusively utilized the SI as a metric to gauge swallowing capability. While the assessment of the impact of sensation on swallowing ability can be carried out through the measurement of swallowing intervals following pharyngeal stimulation, this methodology does not enable the evaluation of the effect of sensation on the central nervous system since SI only reflects the period of muscle activity. As such, it is essential to employ techniques that measure brain function, such as fMRI, to assess the effects of carbonic acid in the triggering of swallowing. Oral stimulation by carbonated water activated the insular cortex, and the activated area differed from the area activated by sour taste stimulation in the insula.^[36]^ Connectivity analysis using fMRI reported that swallowing includes the bidirectional connection between the supplementary motor area and the primary sensorimotor cortex and a 1-way connection from the primary sensorimotor cortex to the insula.^[37]^ Therefore, stimulation by carbonic acid may promote the central triggering of swallowing by activating the insular cortex. Furthermore, pharmacological interventions that utilize inhibitors of carbonic anhydrase, such as acetazolamide, may prove useful in evaluating the impact of carbonic acid. Second, because we recorded repetitive voluntary swallowing, secretion of saliva may have affected the SIs during the pharyngeal stimulation. The resting flow rate of saliva depends on age and sex. One study showed that the average resting whole saliva flow rate was 0.52 mL/minute in young men, 0.41 mL/minute in young women, 0.32 mL/minute in advanced-age men, and 0.25 mL/minute in advanced-age women.^[38]^ In any of these categories, the resting whole saliva flow rate was faster than the solution infusion speed used in the present study. Therefore, to consider the interindividual variation of SIs, it may have been necessary to examine the correlation of the SI with the salivary flow rate. Third, we did not check the tip position of the pharyngeal tube after insertion. The tube tip was positioned 12 cm away from the mandibular central incisor in accordance with previous studies.^[10,12,39]^ In previous studies, the location of the tube tip at a distance of 12 cm from the mandibular incisor was confirmed via endoscopic observation.^[10,39]^ Visual verification demonstrated that the tube tip was situated deeper than the oral fauces both before and after the infusion of the solution in this study. However, it was not confirmed that the tube tip was situated within the laryngopharyngeal region during the experiments. As a result, it is possible that the pharynx may not have been effectively stimulated in some participants. Fourth, the restricted sample size and limited representation of healthy individuals in this study do not allow for the elimination of asymptomatic subjects who might have compromised swallowing function or the influence of age or gender. The selection of participants may have required additional assessments, such as video fluoroscopy, to provide a more comprehensive physiological analysis. The inclusion of video fluoroscopy could have provided further insights into the movement of the epiglottis, penetration-aspiration scale, pharyngeal transit time, or pharyngeal retention in healthy subjects. Aging affects swallowing function by reducing sensory sensitivity.^[40]^ Aviv^[41]^ examined the thresholds for the air pulse discrimination test in the pharyngeal region and found a progressive increase in the discrimination threshold with advancing age. Kamarunas, et al^[42]^ showed that older subjects required a four-fold higher volume than younger subjects in oral liquid volume perception. The decrease in sensory sensitivity in the oropharyngeal region might be risk factors of dysphagia and aspiration. Future studies are needed to investigate these questions and limitations.

It was observed that greater shortening of the SI occurred during repetitive voluntary swallowing tasks when stimulated with carbonated NaCl as compared to NaCl alone. This implies that the sensory input provoked by pharyngeal carbonic acid stimulation is advantageous in enhancing the swallowing reflex. Given that pharyngeal hypesthesia is a considerable risk factor for dysphagia, carbonic acid stimulation of the pharyngeal area could improve dysphagia by inducing reflexive swallowing. The current findings indicating that carbonated water sensation amplifies the capacity for repetitive voluntary swallowing could be a straightforward sensory stimulation-based rehabilitation technique for dysphagia patients, particularly those with decreased pharyngeal sensitivity.

## Acknowledgments

This work was supported by a Grant-in-Aid from the Ministry of Education, Culture, Sports, Science and Technology of Japan (No. 21K11278 to H.F.) and a grant from the Baika Women’s University Project Research Grant (2021-2 to M.T.). The authors thank Angela Morben, DVM, ELS, from Edanz (https://jp.edanz.com/ac) for editing a draft of this manuscript.

## Author contributions

**Conceptualization:** Mika Tsuchiya, Hideyuki Fukami.

**Data curation:** Mika Tsuchiya, Hideyuki Fukami.

**Formal analysis:** Mika Tsuchiya, Chie Omori.

**Funding acquisition:** Mika Tsuchiya, Hideyuki Fukami.

**Investigation:** Mika Tsuchiya, Yumiko Kubo, Naomi Maruyama, Chie Omori.

**Methodology:** Hideyuki Fukami.

**Project administration:** Hideyuki Fukami.

**Resources:** Hideyuki Fukami.

**Supervision:** Hideyuki Fukami.

**Validation:** Mika Tsuchiya, Hideyuki Fukami.

**Writing – original draft:** Mika Tsuchiya, Hideyuki Fukami.

**Writing – review & editing:** Hideyuki Fukami.
